# Superinfection with drug-resistant HIV is rare and does not contribute substantially to therapy failure in a large European cohort

**DOI:** 10.1186/1471-2334-13-537

**Published:** 2013-11-12

**Authors:** István Bartha, Matthias Assel, Peter MA Sloot, Maurizio Zazzi, Carlo Torti, Eugen Schülter, Andrea De Luca, Anders Sönnerborg, Ana B Abecasis, Kristel Van Laethem, Andrea Rosi, Jenny Svärd, Roger Paredes, David AMC van de Vijver, Anne-Mieke Vandamme, Viktor Müller

**Affiliations:** 1 Institute of Biology, Eötvös Loránd University, Budapest, Hungary; 2 School of Life Sciences, École Polytechnique Fédérale De Lausanne, Lausanne, Switzerland; 3 Institute of Microbiology, University Hospital and University of Lausanne, Lausanne, Switzerland; 4 Swiss Institute of Bioinformatics, Lausanne, Switzerland; 5 High Performance Computing Centre, University of Stuttgart, Stuttgart, Germany; 6 Computational Science, The University of Amsterdam, Amsterdam, The Netherlands; 7 Department of Medical Biotechnologies, University of Siena, Siena, Italy; 8 Institute of Infectious and Tropical Diseases, University of Brescia, Brescia, Italy; 9 Unit of Infectious Diseases, Department of Medical and Surgical Sciences, University “Magna Graecia”, Catanzaro, Italy; 10 Institute of Virology, University of Cologne, Cologne, Germany; 11 Institute of Clinical infectious Diseases, Università Cattolica del Sacro Cuore, Roma, Italy; 12 University Division of Infectious Diseases, Siena University Hospital, Siena, Italy; 13 Department of Infectious Diseases, Karolinska Institute, Stockholm, Sweden; 14 Centro de Malária e Outras Doenças Tropicais, Unidade de Microbiologia e Unidade de Saúde Pública e Internacional; Instituto de Higiene e Medicina Tropical, Lisboa, Portugal; 15 Rega Institute for Medical Research, Department of Microbiology and Immunology, KU Leuven, Leuven, Belgium; 16 Fundacions IrsiCaixa I Lluita contra la SIDA, Universitat Autònoma de Barcelona, Badalona, Spain; 17 Erasmus Medical Centre, Erasmus University Rotterdam, Viroscience, Rotterdam, The Netherlands; 18 Research Group of Theoretical Biology and Evolutionary Ecology, Eötvös Loránd University and the Hungarian Academy of Sciences, Budapest, Hungary

**Keywords:** HIV, Superinfection, Transmitted drug resistance, Sequence analysis

## Abstract

**Background:**

Superinfection with drug resistant HIV strains could potentially contribute to compromised therapy in patients initially infected with drug-sensitive virus and receiving antiretroviral therapy. To investigate the importance of this potential route to drug resistance, we developed a bioinformatics pipeline to detect superinfection from routinely collected genotyping data, and assessed whether superinfection contributed to increased drug resistance in a large European cohort of viremic, drug treated patients.

**Methods:**

We used sequence data from routine genotypic tests spanning the protease and partial reverse transcriptase regions in the Virolab and EuResist databases that collated data from five European countries. Superinfection was indicated when sequences of a patient failed to cluster together in phylogenetic trees constructed with selected sets of control sequences. A subset of the indicated cases was validated by re-sequencing *pol* and *env* regions from the original samples.

**Results:**

4425 patients had at least two sequences in the database, with a total of 13816 distinct sequence entries (of which 86% belonged to subtype B). We identified 107 patients with phylogenetic evidence for superinfection. In 14 of these cases, we analyzed newly amplified sequences from the original samples for validation purposes: only 2 cases were verified as superinfections in the repeated analyses, the other 12 cases turned out to involve sample or sequence misidentification. Resistance to drugs used at the time of strain replacement did not change in these two patients. A third case could not be validated by re-sequencing, but was supported as superinfection by an intermediate sequence with high degenerate base pair count within the time frame of strain switching. Drug resistance increased in this single patient.

**Conclusions:**

Routine genotyping data are informative for the detection of HIV superinfection; however, most cases of non-monophyletic clustering in patient phylogenies arise from sample or sequence mix-up rather than from superinfection, which emphasizes the importance of validation. Non-transient superinfection was rare in our mainly treatment experienced cohort, and we found a single case of possible transmitted drug resistance by this route. We therefore conclude that in our large cohort, superinfection with drug resistant HIV did not compromise the efficiency of antiretroviral treatment.

## Background

Superinfection occurs when a patient with established HIV infection is infected with a second viral strain [1], and may have epidemiological and clinical implications in the HIV pandemic. It allows for the recombination of two distinct lineages [2], which may facilitate viral evolution [3-6]. It can also lead to the loss of viral control, if a superinfecting viral strain escapes CD8 immune responses [7], and can accelerate disease progression [8]. Furthermore, an established drug sensitive HIV strain can be invaded by superinfection with a drug resistant virus [9].

Finding at least two distinct viral strains in the samples of the same patient that have not evolved by divergence within that patient implies superinfection. While such a situation may also arise by simultaneous transmission of two distinct viral strains from the same infecting source [1], considering the low number of transmitted viruses [10] such simultaneous transmissions are probably rare, and we assume that most cases of “dual infections” reflect superinfection.

Possible transient superinfections present a further confounding factor for the estimation of the prevalence of superinfection [2]. If the second viral strain is able to grow to low levels only, or it is lost after a short transient period, superinfection may remain undetected with low-sensitivity population sequencing or infrequent sampling. Differences in the sampling strategy may thus explain the large variance in the published estimates of the prevalence of superinfection. Some studies found a low (<5%) prevalence of superinfection either by population sequencing (e.g., [11]) or deep sequencing (e.g., [12,13]). Other studies estimated much higher prevalence, e.g., one study identified 23 superinfections in 58 patients of a US cohort by clonal sequencing of several samples per patient [2]. Jurriaans *et al.* identified two cases in a study population of 14 patients [14], and another study reported five cases in a group of 14 high risk women from Kenya [15].

It is unclear whether an established infection decreases the chance of another virus to invade [16,17]. However, effective antiretroviral treatment (ART) reduces the virus load to very low levels, which results in the gradual restoration of target cell levels and the waning of anti-HIV immunity [18]. This could set the stage for the invasion of a virus that can tolerate the drugs. Superinfection of patients under ART could therefore potentially constitute an important route in the spread of drug resistance mutations, in addition to primary mutation and transmission.

In this study we screened for superinfection a large population of patients, most of whom were under ART and had low, but detectable viral loads. While ongoing ART was probably able to prevent superinfection with virus strains susceptible to the current treatment of each patient, this population was ideal for testing whether superinfection is a major contributor to the spread of highly drug resistant HIV that could compromise therapy in treated patients. ART imposes a constant selection pressure that favours highly drug resistant viruses that can grow under these conditions, and the loss of viral control due to superinfection by a resistant virus would likely result in repeated resistance testing, which would enable the detection of the event in our database. Our detection methods enabled us to detect non-transient superinfection where the invading strain outgrows and replaces the resident viral strain: this is the scenario that has practical relevance for drug treatment. Our primary goals were to estimate the frequency of strain replacement (“successful superinfection”) in the Virolab and EuResist collaborative European databases, and to assess whether superinfection contributes to virologic failure due to acquired drug resistance. We based our analyses on routinely collected genotypic data, which have been shown to allow for the detection of superinfection [19].

## Methods

### Ethics statements

All study participants provided written informed consents. Ethical approvals were obtained from the local ethical committees: the Regional Ethical Review Board in Stockholm (2005/1167–31/3), the Ethics Committee of the Catholic University of Rome, the Ethics Committee of the University Clinic Cologne, the Belgian Ethical Committee (B32220072107) and the Ethical Committee of the Spedali Civili di Brescia. The study was conducted in accordance with the declaration of Helsinki.

### Patients and sequences

We analyzed the joint database of the EuResist and Virolab consortia, which included clinical and demographic data, treatment history and sequence data from genotyping assays collected at clinical centres in Belgium, Germany, Italy, Spain and Sweden. Data from the individual data centres were stored in independent instances of the RegaDB data scheme [20] and virtualized into a single collaborative database. We included data from patients from whom sequences were available from a minimum of two distinct time points. We obtained 13816 sequences from 4425 patients who had at least two sequences from distinct time points in the Virolab/EuResist database. The sequences spanned the PR and partial RT regions of the *pol* gene of the viral genome [21,22].

### Phylogenetic software and substitution model

We used RAxML v7.2 [23] and MrBayes v3.1.8 [24] to reconstruct phylogenetic trees. A GTR substitution model was assumed in both cases (based on the advised protocol for RAxML, and also for MrBayes to obtain consistent results). In the RAxML analyses, branch support values were computed based on bootstrap replicates (-autoMR method of RAxML), while posterior probabilities were used with MrBayes. In the case of MrBayes 10,000,000 generations were simulated on 4 chains of 3 simultaneous runs, with 10,000 steps discarded as burnins. We used PAUP [25] to calculate maximum likelihood sequence distances under the GTR model without building a phylogenetic tree.

### Subtyping

HIV subtype was determined with the Rega v2 subtyping tool [26,27].

### Detecting superinfection

**Figure 1:**
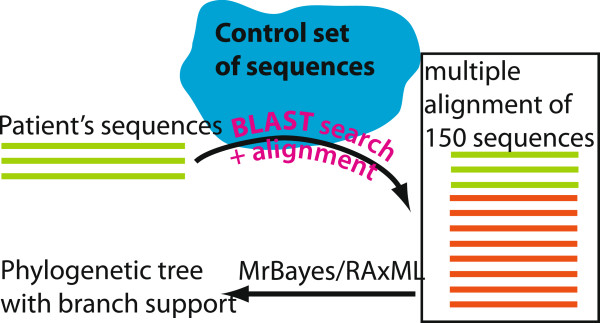
**Scheme of the analysis pipeline for detecting HIV dual infection.** We assembled a control sequence set for each patient by selecting, from a large sequence dataset, the highest ranking BLAST results (closest sequence matches) to each sequence of the patient, such that the total set included 150 sequences, and each sequence of the patient was matched with the same number of control sequences. Phylogenetic trees were then constructed and analyzed for each sequence set, one tree per patient.

To decide whether a patient had been infected by two distinct viral strains we constructed a phylogenetic tree with all sequences of the patient and a fixed number of control sequences. We considered an individual to be superinfected if his/her own HIV sequences failed to form a monophyletic cluster in the tree, with at least a threshold number of control sequences clustering together with the sequences of the patient (see below Evaluating Phylogenetic Trees). Following the procedure in [28] (see Figure 1), control sequences were selected by a BLAST search [29] using a local BLAST database assembled from the set of all available HIV sequences that spanned the same genomic region (partial *pol* or *env*) (see below Local BLAST database). We constructed a control sequence set for each patient by submitting each sequence of the patient to a BLAST search and selecting from the BLAST result list (ordered in descending similarity score) a fixed number of the most similar sequences of appropriate length. All sequences of the patient were pooled in a single analysis and topped up to a fixed total number (e.g. 150) by adding the same number of matched control sequences per patient sequence. The set of control sequences did not contain duplications. Individual phylogenetic trees per patient were then constructed using the patient strains and its control set, and evaluated as described below.

#### Local BLAST database

We downloaded 44244 partial HIV-1 B sequences from the Los Alamos HIV Database (http://www.hiv.lanl.gov/) that spanned HXB2 coordinates 2253-3290, and merged them with 26130 sequences spanning the same region in the Virolab/ EUResist database. Note that the latter count included also sequences from patients who were part of the Virolab/EUResist database but were not eligible for the detection of superinfection, because only one sequence was available from them. Sequence alignment was done by Muscle v3.8.31 [30]. *Pol* sequences were aligned by Muscle in batches of 1000 sequences, then these partial alignments were merged using the HXB2 reference sequence as a guide. Codons associated with drug resistance were removed based on the Stanford HIV Drug Resistance Database (as of 2009) [31]; gap-stripping (with a threshold of 50%) removed all insertions relative to the reference sequence. A local BLAST database was compiled from the alignment. Another BLAST database was built from publicly available *env* sequences (13725 sequences downloaded from the Los Alamos HIV Database) for the validation procedure that involved also *env*. *env* sequence alignment was constructed with translated amino acid sequences using Muscle v3.8.31 [30]. Sequence translation was done by transeq [32]; frame-shifts were corrected manually.

#### Evaluating phylogenetic trees for superinfection

**Figure 2:**
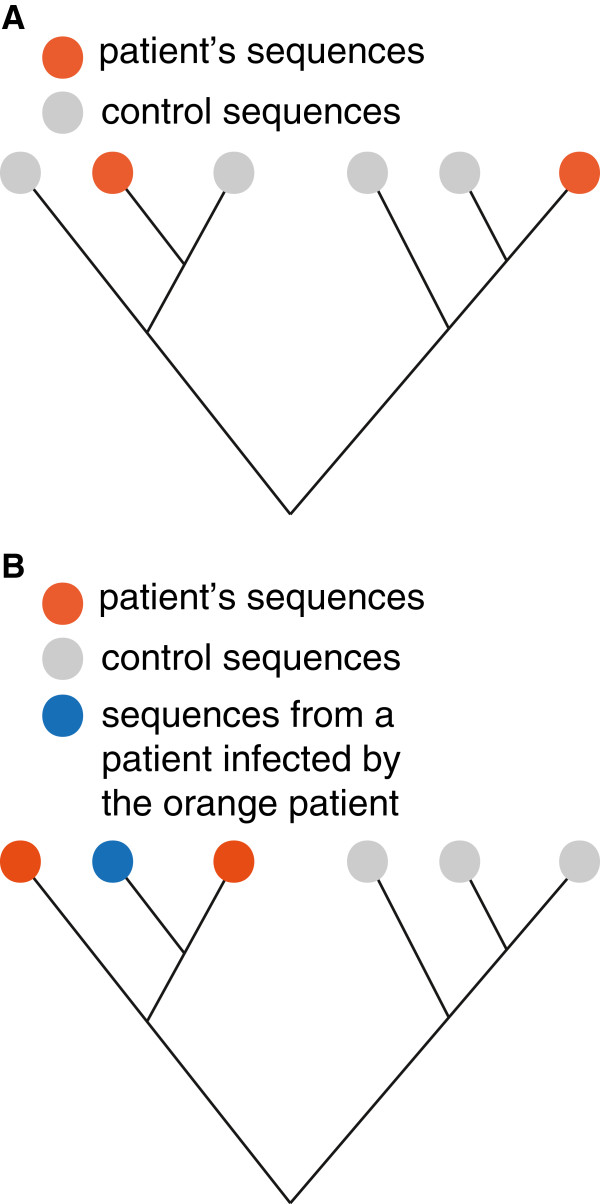
**Scheme of phylogenetic trees representing superinfection or transmission cluster.** Orange nodes denote sequences from the individual under examination; grey nodes represent control sequences. Panel **A** illustrates a case of superinfection, while Panel **B** illustrates a transmission cluster. The sequences of the patient under study fail to form a monophyletic cluster in both cases; however, the number of control sequences in the smallest subtree that includes the sequences of the patient tends to be much greater in the case of superinfection (see main text).

We developed Ruby scripts (available in Additional file 1) to evaluate whether the sequences of a patient form a monophyletic cluster in the phylogenetic tree constructed from the HIV sequences of the patient and the control sequences selected by BLAST. We collapsed all internal nodes that had low support to polytomic nodes: nodes with a minimum bootstrap support value of 60 or a minimum posterior probability of 0.95 were retained in the trees constructed with RAxML and MrBayes, respectively. A tree was considered to reflect putative superinfection when i) at least two sequences of the patient clustered closer with control sequences than with each other (Figure 2) and ii) the size of the minimal subtree that contained all sequences of the patient was larger than 50, based on Additional file 2. We applied the strict first criterion to ensure that weakly supported misclustered sequences could not induce a false positive finding. By applying the second criterion we hypothesized that a small number of control sequences in the subtree is more likely to reflect a transmission cluster (possibly originating from the patient under study) than a genuine case of superinfection. The distribution of cluster sizes is depicted in Additional file 2. The strong bimodality of the distribution indicated a gap between potential transmission clusters and potential superinfections, with an obvious cut-off at a cluster size of 50 control sequences. We therefore classified the cases with <50 control sequences in the minimal subtree as putative transmission clusters. Some such cases may also have arisen if sequences of the same patient occurred in the database under different identifiers, either from sample mix-up or from GenBank. This additional criterion also helped to minimise false positive results arising from ambiguously clustered sequences.

### Experimental validation of putative superinfection cases

To validate several cases of putative superinfection, we performed fresh sample amplification and population sequencing on selected archived samples, involving either the same *pol* region as in the original sequence data, or the C2-C4 region of *env* (HXB2 positions 7021-7560) from the samples dating from just before and just after the putative event of superinfection [9,33-35]. The newly obtained sequences were then checked for superinfection by the same analysis pipeline as in the original analyses. To assess whether the newly obtained sequences cluster together with (any of) the original sequences of the patient, we included also the original sequences in the alignment used to infer the phylogenetic trees.

### Quantitation of drug resistance (drug susceptibility scores)

We used the Stanford HIVdb Sequence Analysis web service (http://hivdb.stanford.edu, [31]) for the estimation of drug resistance. We report Stanford resistance classes based on Stanford Scores (0-9: S; 10-14: PL; 15-29: LL; 30-59: R ; 60+: HR). A higher score means a more resistant virus strain, with weaker virologic response to drug treatment.

## Results

Based on our eligibility criteria 13816 sequences from 4425 patients were retained for the analysis. Most sequences spanned part of *pol* (protease (PR) and partial reverse transcriptase (RT) genes), with an average length of 1026 nucleotides. The median sequence count was 2 (range: 2-29) per patient.

3653 participants received ART during the follow-up period. For 772 patients treatment information was not available. 1123 individuals (25%) were heterosexual, 910 (20%) men having sex with men, 1000 (22%) intravenous drug users, 1236 (27%) unknown and 156 (3%) were in other risk groups. 1308 (29%) individuals were female. For 3878 participants we had access to at least one viral load measurement, 3552 of those had at least one measurement above 10000 copies/ml. Viral load measurements in the database had a median of 3.3 log10 copies/ml (IQR: 1.9-4.4 log10 copies/ml). CD4 measurements in the database had a median of 349 cells/ *μl* (IQR: 208-521 cells/ *μl*). The first CD4 or viral load test had a median date of 1997-11-17 (IQR: 1995-01-30 - 2001-10-15). Median age at the earliest data point was 34.8 years (IQR: 29.3 - 40.9 years). Time between the earliest and latest sample of a patient had a median of 756 days (IQR: 367-1497 days). 86% of the HIV sequences belonged to subtype B (Additional file 3), and 86% (3814/4425) of the patients were infected with this subtype. 19.2% (2655/13816) of the sequences were resistant to all three drug classes (PI, NRTI, NNRTI) associated with the available genomic region, and 28.2% (1250/4425) of the patients had at least one such sequence, indicating a highly drug-experienced cohort.

### Detection of superinfection based on phylogenetic analysis

The detection method relies on the analysis of phylogenetic trees constructed from the sequences of a patient and a set of control sequences matched to the patient’s sequences by sequence similarity from a large database (see below). In such a tree, if the minimal subtree that includes all sequences of an individual includes also one or more control sequences, then either i) the most recent common ancestor of the sequences was not located in the patient, which indicates superinfection with a distinct lineage (Figure 2A) or ii) the control sequences are derived from a transmission cluster that originated from the patient (Figure 2B).

In our analysis we searched for cases of super (or dual) infection by the following two criteria: first, at least two sequences of the patient had to form a strongly supported monophyletic cluster with unrelated control sequences. That is, at least two strongly supported clusters had to be present in the tree that each contained at least one sequence taken from the patient under investigation, together with control sequences (see Methods, and Figure 2A). This choice constitutes a stringent criterion for the polyphyly of the patient’s sequences.

Second, the minimal subtree that contained all sequences of the patient had to include at least 50 unrelated control sequences. Given the sparse sampling of sequences in the epidemic, transmission clusters are likely to be represented by small numbers of sequences; the exact threshold was set based on the distribution of the cluster sizes (see Methods and Additional file 2). Furthermore, this criterion also excludes false positive results that arise when a sequence of the same patient is included in the control set from anonymous or mislabeled records in the databases.

Building a reliable phylogenetic tree with a very large number of sequences is computationally intensive. Therefore we performed a series of preliminary analyses to optimize the analysis pipeline of phylogeny-based detection of superinfection in a large number of patients (see Additional files 4 and 5).

For each of the 4425 patients we built a phylogenetic tree with a set consisting of a fixed number of control sequences. We selected the set of control sequences for each patient from a massive local BLAST database of 70374 sequences such that each sequence of a patient was matched and grouped with an equal number of similar sequences (see Methods and Figure 1). We considered using RAxML [23], a fast and reliable implementation of the maximum likelihood phylogenetic approach and MrBayes [24] which implements the much slower but more exhaustive Bayesian approach. We first performed exploratory analyses to compare the performance of Bayesian (MrBayes) and maximum likelihood (RAxML) methods on a small number (n=170) of patients, and to calibrate the optimal support thresholds for both methods and the optimal size of the control sequence set. The set of 170 patients for the calibration runs was selected by preliminary analyses detailed in Additional file 4, and was enriched in putative cases of superinfection.

Based on the benchmarks tests performed on these 170 patients with RAxML and MrBayes we found that the number of cases identified as non-monophyletic infection (either superinfection or transmission cluster) declines as the threshold for branch support is increased; however, the two methods could be calibrated to identify consistent sets of patients (Table 1). E.g., the subset identified as non-monophyletic with the widely used threshold of 0.95 posterior probability with MrBayes could be reproduced using a threshold bootstrap support of 0.6 in RAxML. We therefore concluded that the faster maximum likelihood based RAxML method ensured sufficient sensitivity for our analyses. To find the optimal size of control sequences in the analyses, we next performed both MrBayes and RAxML analyses with tree sizes of 20, 50, 150 and 250 sequences on the same subset of the data (170 patients). There was no apparent gain in the number of suspected patients with increasing tree size (Additional file 6), and the identity of the suspected patients was stable across different tree sizes (Table 2). This indicates that, using the BLAST based method to select control sequences from a massive sequence database, relatively small trees are sufficient to identify the phylogenetic footprint of superinfection reliably.

**Table 1:** Comparison of methods and calibration of support threshold for cluster based prediction

**MB**	**RAxML**	**Intersect**	**RAxML**	**MB**	**RAxML**	**MB**
			**only**	**only**	**sum**	**sum**
0.7	0.6	70	2	4	72	74
0.7	0.7	63	2	11	65	74
0.7	0.95	40	2	34	42	74
0.8	0.6	70	2	3	72	73
0.8	0.7	63	2	10	65	73
0.8	0.95	40	2	33	42	73
● 0.95	0.6	69	3	2	72	71
0.95	0.7	62	3	9	65	71
0.95	0.95	40	2	31	42	71

Comparison of results obtained by RAxML and MrBayes at varied thresholds for branch support or posterior probability used in the collapsing of phylogenetic trees. Numbers of patients with non-monophyletic sequences for given threshold values are shown in the cells. Tree size was 150 in all cases (i.e. the tree was constructed from an alignment of 150 sequences). The analyses were performed on a set of 170 patients derived from a preliminary analysis and enriched in superinfection cases. Full circle indicates the choice of parameters for the final analyses.

**Table 2:** Comparison of methods and calibrating the size of the phylogenetic tree

	**RAxML 150 0.6**	**MrBayes 150 0.95**	**RAxML 20 0.6**	**MrBayes 20 0.95**	**RAxML 250 0.6**	**RAxML 50 0.6**	**MrBayes 50 0.95**
RAxML 150 0.6	72						
MrBayes 150 0.95	69	71					
RAxML 20 0.6	67	66	70				
MrBayes 20 0.95	61	62	62	63			
RAxML 250 0.6	69	67	66	59	71		
RAxML 50 0.6	69	66	65	59	67	69	
MrBayes 50 0.95	67	68	64	62	65	66	69

The table shows the number of patients indicated with non-monophyletic infection using the settings of both the row and the column header of a cell, from a preliminary analysis of 170 individuals. Headers show the phylogenetic software used for tree reconstruction, the size of the tree and the support threshold used when evaluating the trees.

Informed by these results, in the final analysis of all 4425 patients, we used RAxML (for faster speed over MrBayes) with a threshold bootstrap support of 0.6 (to obtain results consistent with MrBayes and a threshold of 0.95 posterior probability) and a tree size of 150 (which still allowed fast phylogenetic reconstruction). Thus, each tree was built from 150 sequences, including all sequences of a single patient, and complemented with control sequences to reach a total number of 150 sequences. The sequences of 201 patients failed to form a within-patient monophyletic cluster, with control sequences clustering nested within the patient’s sequences. Out of these, 107 were suspected for superinfection and in 94 cases the sequences of the patient belonged to a closely related transmission cluster (size of minimal subtree ≤ 50, see Methods). One of the 107 putative superinfection cases turned out to be a false positive arising from mixing the sequence reads from the patient strain and a lab strain, and was excluded from further analysis.

### Validation

To validate our results, we had the possibility to re-sequence stored samples from 14 putative superinfection cases and 4 controls. In the cases of putative superinfections, we attempted to analyze pairs of stored samples dated as close as possible to and spanning the putative event of superinfection. For two patients, the original sample after the suspected superinfection event was not available, we therefore used a sample obtained shortly after the original sample. We amplified and (re-) sequenced partial *pol* in 6 cases, the C2-C4 region of *env* in 4 cases, and both regions in 8 cases. Attempts to amplify sequences for several other cases failed due to the degradation of old samples. Analyses of the new sequences confirmed two out of 14 cases of superinfection (Table 3). In 9 out of 14 putative superinfection cases, the new *pol* sequences of the patient formed a monophyletic cluster, indicating no evidence for superinfecton.

**Table 3:** Validation of putative superinfection cases

**Patient**	**Origin/Follow-up**	**Sex**	**Risk**	**Sub-**	**Validated**	**Result of**	**Identified**	**Estimated**
			**group**	**types**	**gene**	**validation**	**mixed-up**	**date of**
							**sample**	**event**
Case1	-/Spain	M	HET	B,*	POL	monophyletic	10/26/2000	
Case2	Spain/Spain	M	HET	B	POL ENV	monophyletic	02/19/2004	
Case3	Spain/Spain	F	HET	B,G	POL ENV	monophyletic^1^	04/19/2004	
Case4	Sweden/Sweden	M	MSM	B	POL ENV	monophyletic	05/05/2004	
Case5	Eritrea/Sweden	M	NA	C	ENV	monophyletic^2^		
Case6	-/Belgium	M	HET	*	ENV	monophyletic		
Case7	-/Italy	M	MSM	*,B	POL	monophyletic	01/25/2006	
Case8	Italy/Italy	M	VERT	B	POL ENV	cluster of closely related samples		
Case9	-/Italy	M	NA	B	POL ENV	monophyletic	19/07/1999	
Case10	-/Italy	M	NA	B	POL	monophyletic	04/20/1998	
Case11	Italy/Italy	F	HET	B	POL	monophyletic	09/10/1997	
Case12	Italy/Italy	F	IVDU	B	POL	monophyletic	11/24/1998	
● Case13	-/Italy	F	HET	B	POL ENV	POL: non monophyletic ENV: non monophyletic but fails criteria (low support)		Jan 98- May 98
●Case14	-/Italy	M	IVDU	B	POL ENV	POL: non monophyletic ENV: non monophyletic but fails criteria (low support)		Nov 97- Nov 98
Control1	Spain/Spain	M	MSM	*	POL ENV	monophyletic		
Control2	Spain/Spain	M	HET	B	POL ENV	monophyletic		
Control3	-/Spain	M	HET	*	ENV	monophyletic		
Control4	-/Belgium	M	HET	CRF 02_AG	ENV	monophyletic		

Results of the validation of 14 cases of putative superinfection and 4 controls. Samples where thawed, amplified and *pol* and *env* regions where sequenced as described in the Methods. Full circles indicate confirmed superinfection cases. Notes: ^1^the sample before the superinfection event was unavailable, an alternative was used; ^2^neither of the original samples were available and two alternatives were used. An asterisk (*) indicates that subtype could not be determined uniquely. Abbreviations: IVDU - intravenous drug users; HET - heterosexuals; MSM - men having sex with men; VERT - vertical transmission; NA - unknown; F - Female; M - Male.

In all of these cases, the new sequences clustered together closely with one of the original clusters of the patient, indicating that the original sequence that clustered separately did not come from the patient, but was involved in sample or sequence mix-up. In three of the 9 cases, the C2-C4 region of *env* could also be amplified from the paired samples, and the analyses yielded consistent results (no evidence for superinfection).

In two cases, only *env* could be sequenced from both original samples: the analyses indicated no evidence for superinfection; this data, however, did not allow us to determine which of the original *pol* sequences was erroneously recorded for the patient.

Finally, in one case the new analysis found a reduced number of control sequences in the minimum subtree of the patient, indicating a probable transmission cluster of closely related sequences (all from the same country), rather than superinfection. A re-analysis of the original sequences of this patient (with the original set of matched control sequences) by MrBayes confirmed that the initial RAxML analysis misplaced some of the control sequences that apparently clustered together with the patient’s own. Newly amplified *env* sequences of this patient formed a monophyletic cluster.

In the two confirmed superinfection cases, the analysis of both *pol* and *env* indicated evidence for superinfection, although branch support was low for *env*. From one of these patients we had only two sequences, i.e., both the initial and the putative superinfecting strain were only present in a single sample. This patient had persistent low CD4 count (<50 cells per *μ*L) in the years preceding and following the inferred superinfection event. We had 7 sequences from the other patient, of which 6 clustered together. A single sequence represented a divergent lineage, and it was both preceded and followed by samples that yielded sequences of the larger cluster, which probably indicates a case of transient superinfection. There was an abrupt drop in the CD4 count (from 430 to 125 cells per *μ*L) in the one-year interval between the two samples that indicated the strain switching, and this loss was not restored when the original strain re-appeared in the subsequent sample points. Importantly, strain switching (superinfection) was not associated with increasing drug resistance scores against the drugs that were administered at the time of strain switching in either of the patients (Table 4). Clinical and demographic parameters, treatment history and drug susceptibility scores of these two patients are shown in Additional file 7.

**Table 4:** Drug resistance interpretation for the three superinfection cases

**Regime**	**Case13**		**Case14**		**DBC+**	
	Resident strain	Superinfecting strain	Resident strain	Superinfecting strain	Resident strain	Superinfecting strain
3TC	HR	HR	PL	S	●S	HR
ABC	HR	LL	R	S	S	●HR
ATV/r	S	LL	S	S	S	S
AZT	HR	R	●HR	●LL	●PL	HR
D4T	●HR	●R	HR	PL	LL	●HR
DDI	●HR	PL	●R	●S	PL	HR
DLV	S	S	S	S	S	S
DRV/r	S	S	S	S	S	S
EFV	S	●S	S	S	S	S
ETR	S	S	S	S	S	S
EVG	S	S	S	S	S	S
FPV/r	S	LL	S	S	S	S
FTC	HR	HR	PL	S	S	HR
IDV/r	S	R	●S	●S	S	S
LPV/r	S	PL	S	S	S	S
NFV	●S	●HR	S	S	S	S
NVP	S	S	S	S	S	S
RAL	S	S	S	S	S	S
SQV/r	S	R	S	S	S	S
TDF	R	PL	R	S	PL	●LL
TPV/r	S	S	S	S	S	S

Drug resistance (based on http://hivdb.stanford.edu) in the two patients with validated superinfection and the patient suspected for superinfection based on high degenerate base code (DBC) count. Drug resistance interpretation is shown for the samples preceding and following the suspected superinfection event; bullets indicate the drugs administered in the sample interval. In Case 13, resistance against two of the drugs decreased, while resistance against one drug increased concomitant with superinfection; in Case 14, resistance against the current drugs decreased; in the DBC+ case, resistance against both drugs increased. None of the cases involved currently recommended drug regimens. S: Susceptible, PL: Potential low-level resistance, LL: low-level resistance, R: resistance, HR: high resistance.

None of the four control cases yielded evidence for superinfection. Sequence alignments of both the original and the newly generated sequences of the 18 patients involved in the validation procedure and their control sequences are attached in Additional file 8.

### Detection based on degenerate base pair count

Finally, following the method of Cornelissen *et al.*[33] we also counted the number of degenerate base codes (DBC) in all available sequences. A high DBC count may indicate that a mixture of two divergent virus strains was present in the patient at the time of sampling: using clonal sequencing, Cornelissen *et al.* found that a DBC count of 45 in routine genotyping sequences (such as used in the present study) is associated with dual infection in about 73% of the cases. In our study, 79 patients had at least one sequence with a DBC count higher than or equal to 45 (5 patients had two, and one patient had three such sequences). Of these 79 patients, six were suspected for superinfection based on our phylogenetic analysis with RAxML. One case (Case2 in Table 3) was subjected to validation, and the result indicated sample mix-up as the source of the false positive superinfection signal: notably, the sample yielding the high DBC count was the sample involved in a mix-up. The analyses of both *pol* and *env* showed strong monophyletic clustering with the newly amplified sequences of this patient.

In 23 of the 79 cases the sequence with high DBC count was flanked by two sequences with low DBC counts (indicating homogeneous viral populations). In one of these cases (which we could not validate by fresh sample amplification) the sequence with high DBC count was preceded and followed by two sequences that clustered independently from each other. This situation is highly suggestive of successful superinfection (strain replacement), with the high DBC count intermediate sample representing a transitory stage when a mixture of both strains was present. In this patient drug resistance scores against the drug regimen administered at the time increased considerably (Table 4), and there was a temporary episode of detectable viremia around the suspected time of superinfection (Additional file 7). In the remaining 22 cases where we had samples from both before and after the sequence with high DBC count, all sequences clustered together, indicating that if superinfection was indeed responsible for the sample with high DBC count, it must have been transitory and the original strain persisted.

## Discussion

HIV superinfection can lead to clinical complications [8], intermittent viral rebounds or loss of viral control [7]. However, its population level prevalence and its clinical implications are less well understood, with a large variation in the estimate of the frequency of superinfection in various studies. Our study is the largest to date to estimate the population level frequency of non-transient HIV superinfection among patients under antiretroviral treatment, and to assess its impact on the failure of therapy due to drug resistance.

**Figure 3:**
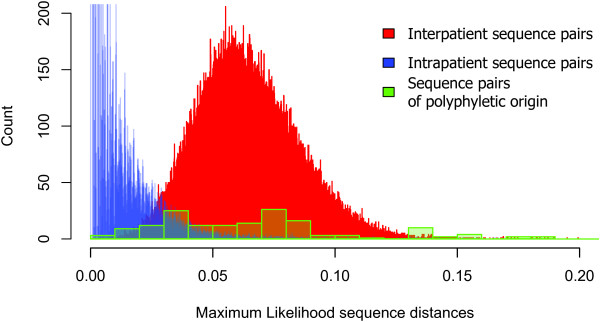
**Distributions of pairwise sequence distance for sequence pairs drawn from the same patient (intrapatient) or from different patients (interpatients), and for sequence pairs of polyphyletic origin from putative superinfected patients.** The distribution of the distances for 80.000 randomly chosen sequence pairs of different patients is shown in red. Intrapatient sequence distances are shown in blue (truncated above 200 to optimize the scale for comparison). The green overlaid histogram depicts polyphyletic sequence pairs from potentially superinfected patients (identified from the initial phylogenetic analysis). There is no clear cutoff for the distribution of intrapatient distances.

We developed an analysis pipeline that allowed us to detect dual infection in large databases by leveraging modern and reliable approaches of phylogenetic inference. This method takes advantage of the broad availability of HIV sequence data both in public and private databases, as the detection of divergent lineages requires a sufficiently large sample of genotypes from the underlying epidemic. We had rigorously analysed the choices in the bioinformatics pipeline before we settled with the method that had the speed and accuracy to cope with the large number of samples in the analysis (see Additional file 4 and Results). The use of phylogenetic reconstruction is justified by the overlap of the distributions of pairwise sequence distances among sequence pairs drawn from the same patient, from different patients of the same subtype, and sequence pairs of polyphyletic origin from potentially superinfected patients (Figure 3).

We note that our criteria of dual infection were more stringent than requiring (potentially weak) non-monophyletic clustering of a patient’s sequences. We classified the cases as dual infection if the phylogenetic tree incorporated at least two strongly supported clusters that each contained at least one sequence from the patient under investigation and control sequences; furthermore, we required a threshold number of control sequences interspersed within the smallest subtree that included all sequences of a patient. This procedure prevents possible false positive results arising from weakly supported clusters, and distinguishes between transmission clusters and superinfection cases. We also engaged in a thorough validation of putative superinfection cases (identified on the basis of our rigorous phylogenetic criteria) by re-analyzing samples from a subset of the patients involved. We thus opted for high specificity at the cost of sensitivity. We are not aware of any large scale study taking this approach.

The initial phylogenetic analysis suggested superinfection in 107 out of 4425 patients. However, re-analysis of stored samples for 14 of these cases confirmed only two out of 14 cases of putative superinfection. Importantly, we used the same bioinformatics pipeline in the original analysis and in the validation procedure: that is, the failure to validate most cases of putative superinfection did not arise from the (lack of) sensitivity of the original phylogenetics analysis, but due to erroneous entries (sequence or sample mix-up) in the original database. While the low number of cases available for validation do not allow us to derive reliable estimates on the frequency of superinfection in this study population, and the rigorous criteria of our detection method may have missed some genuine superinfection cases, the frequency is likely to be very low. Even 107/4425 cases would imply only about 2.4% prevalence, and most of these cases seem to have originated from sample mix-up, according to our validation analyses. Ongoing therapy prevents superinfection with virus strains susceptible to the current treatment of each patient, which has probably also contributed to the low frequency of non-transient superinfections.

Although this result may not be informative of the frequency of superinfection in untreated patients, it indicates that non-transient superinfection with strain replacement occurs rarely during ART. In the whole analysis, we found a single case (inferred from degenerate base code count and the pattern of consecutive sequences) where a superinfecting virus seems to have reduced susceptibility against drugs administered to the patient at the time. We have also compared the change in drug resistance scores at the inferred superinfection events in the patients whose results we were not able to validate by re-requencing versus all adjacent sequence pairs of the patients with no evidence for superinfection, and the difference was not significant (p=0.48, Wilcoxon test). This indicates that, although the true number of genuine superinfection cases cannot be known in the unvalidated set, these cases did not contribute substantially to increasing drug resistance.

Our analysis would have been unable to detect superinfecting virus strains that grew to low levels only or that were outcompeted by the original strain between the samples subjected to routine sequencing. While this may have resulted in an underestimation of superinfection in a strict sense, such low-level or transient superinfections that do not affect the dominant virus population can have only limited effect on the clinical outcome. Furthermore, some evidence indicates that superinfections may occur primarily in the early stages of the initial infection (reviewed in [1]), when routine genotypic data are typically not yet available. Such superinfections would go undetected in our analyses; however, superinfection with highly drug resistant viruses (that would result in subsequent therapy failure) is expected to be rare in the absence of ART, given the reduced replicative capacity of such strains [36] and the overall very low frequency of such viruses in untreated individuals [37]. Our results thus indicate that superinfection does not contribute substantially to the spread of drug resistance in treated individuals and does not compromise the efficacy of therapy at the population level in this European cohort.

Because our analysis depended on sequences obtained in routine genotypic assays, the requirement for sequences from at least two time points per patient may have resulted in biased sampling of the study population. A second (third etc.) genotypic assay may often be motivated by the loss of viral suppression (to test for the emergence of drug resistance), while patients with suppressed virus load are less likely to be subjected to subsequent tests. However, such a bias would actually facilitate the detection of therapy failure due to drug resistance transmitted by superinfection. The loss of virus suppression would have motivated genotypic testing and such cases would therefore have been included in our database with high probability. Furthermore, the majority (82%) of patients in our cohort were under ART, which provides selective advantage to highly resistant virus strains that can tolerate the drugs, and thereby facilitate superinfection by such viruses. We therefore conclude that our cohort of treated patients comprises an ideal sentinel cohort for detecting superinfections involving the transmission of highly resistant HIV-1 strains that could compromise the efficacy of drug treatment. The lack of evidence for the transmission of drug resistance by superinfection in this population of treated patients strongly indicates that this route of transmission does not contribute substantially to the failure of antiretroviral therapy in the HIV epidemic.

The lack of superinfection with drug resistant strains is probably explained by the reduced replicative capacity of strains with high level drug resistance [36]. The transmission of multidrug-resistant HIV-1 is very rare also in terms of primary transmission to untreated individuals [37]. Low replicative capacity may actually allow for the replacement of virus strains with major drug resistance mutations if superinfection with fitter less resistant strains occurs in the absence of therapy [38,39]. In contrast, viral strains with limited drug resistance may have a replicative capacity close to wild-type (as demonstrated by some primary transmission to uninfected individuals [40]); however, such limited drug resistance is probably not sufficient to allow virus replication under combination ART, and ongoing therapy probably prevented superinfection with partially drug resistant (or drug susceptible) viruses in our cohort. Yet it has to be noted that major drug resistance mutations may revert, and any remaining minor drug resistance mutations may convey wild-type or even increased fitness to the strain [41,42], which would make it less likely to be replaced by superinfection.

The number of degenerate base pairs is indicative of the co-existence of two distinct virus strains at the time point of the sample involved [33], which may or may not be followed by strain replacement. In our study, available sequence data allowed us to test 23 cases with a DBC count ≥45 by phylogenetic analyses of both preceding and subsequent samples. Of these, strain replacement following the high DBC count sample could only be demonstrated in a single case, which suggests that the majority (22/23 or 96%) of superinfection events may be transient, consistent with previous results [2]. However, transient superinfection is unlikely to contribute to transmitted drug resistance. We note here that a high DBC count may result in discarding the sequence at the quality control stage of the sequencing procedure. While there was no systematic filtering applied at our data centres, some such sequences may have been discarded by individual judgement. Our database of routinely collected HIV-sequences may therefore not be an unbiased sample of the whole population with respect to degenerate base pair counts.

Our cluster based detection method did not differentiate between superinfection and dual infection arising from the simultaneous transmission of two divergent virus strains in a single transmission event [9]. However, the low number of transmitted virus particles [10] implies that such simultaneous transmissions are probably rare. Moreover, even simultaneous transmission could transmit two divergent virus strains only if the infecting individual harboured two strains ultimately derived from different sources, and both at a sufficient concentration to allow transmission. Such a combination of rare events seems extremely unlikely, and we therefore argue that most cases of dual infections probably reflect superinfection.

We were able to detect superinfection (strain replacement) on the basis of partial *pol* sequences [19,43]. Some cases of superinfection may have resulted in a new recombinant of the resident and the superinfecting strain that outcompeted both original strains [44]. Our analysis would have detected such cases only if the region of *pol* available was derived from the superinfecting virus. Reliable detection of recombinant forms arising from superinfection would require full genome sequencing.

Finally, we note that our method revealed a high number of sample mix-ups, and also one case where a recombinant sequence was formed through misassembling sequence reads from a patient’s sample and the control lab strain. Almost all data centres were involved in at least one sample mix-up case, which sheds light on the ubiquity of the issue. This result indicates the need for validation by re-sequencing in the detection of superinfection, and the need to sequence at least two samples in court cases that use HIV sequence analysis as sources of evidence.

## Conclusions

We conclude that routine genotyping data are useful for the detection of superinfection; however, extensive validation is required to exclude sample or sequence mix-up or contamination, which may be the most likely source of divergent virus sequences assigned to the samples of the same patient. Our study population (mainly therapy experienced patients with chronic infection) was ideal for detecting the transmission of highly drug resistant HIV-1 strains by superinfection. The lack of evidence in this population thus indicates that this potential route is unlikely to contribute to therapy failure by drug resistance.

## Competing interests

The authors declare that they have no competing interests.

## Authors’ contributions

VM conceived and supervised the study. VM and IB designed the analyses, aided by expert advice from AMV and ABA. IB performed the data analyses. MZ, CT, ES, AdL, AS, AMV, RP, DvdV and KVL, contributed data. PS organized the Virolab resource. MA managed the joint database and extracted the data. AR, JS sequenced stored samples for validation. IB, VM wrote the manuscript. All authors contributed to the design of the study and the final form of the manuscript. All authors read and approved the final manuscript.

## Pre-publication history

The pre-publication history for this paper can be accessed here:

http://www.biomedcentral.com/1471-2334/13/537/prepub

## Supplementary Material

Additional file 1 **Software for evaluating phylogenetic trees with respect to superinfection.** The archive contains Ruby scripts that perform the evaluation of a phylogenetic tree. See README in the archive for details. Click here for file

Additional file 2 **The distribution of the number of control sequences clustering together with the patient’s own sequences in those patients whose sequences failed to form a monophyletic cluster.** The number of clustering control sequences was either small (<20) or large (>50). We hypothesized that the former case is more likely to represent a transmission cluster, and used the criterion for the latter case in the identification of superinfection. Phylogenetic trees were constructed from sets of 150 sequences. Click here for file

Additional file 3 **Table - The distribution of HIV sequences by subtype.** Subtyping was carried out by the REGA subtyping tool [26,27]. Click here for file

Additional file 4 Additional Documentation - Supplementary text describing the verification of our bioinformatics pipeline and the approaches that did not succeed. Click here for file

Additional file 5 Figure - Chart of the whole phylogeny based analysis including the preliminary analyses. Click here for file

Additional file 6 **The detection of superinfection is robust with respect to the number of control sequences in the trees.** We performed both MrBayes and RAxML analyses with tree sizes of 20, 50, 150 and (only for RAxML) 250 sequences on a subset of the data (170 patients).
Branches with a minimum support value of 60 or a minimum posterior probability of 0.95 were retained in the trees constructed with RAxML and MrBayes, respectively. The number of suspected patients was robust with respect to tree size. Click here for file

Additional file 7 **Clinical parameters, treatment history and drug susceptibility scores of the two confirmed superinfection cases and of the putative superinfection case with high degenerative base count.** The archive contains Excel tables with treatment history, CD4 cell counts, viral load measurements and drug susceptibility scores per sample. Click here for file

Additional file 8 **Sequence sets - Sets of case and control sequences for the samples of the 18 validated patients.** Fasta files contain the alignments used to infer superinfection in each validated patient. The sequences from the patient under investigation are indicated in the fasta header lines. Click here for file
