# Supplementary material for: Superinfection with drug-resistant HIV is rare and does not contribute substantially to therapy failure in a large European cohort
Source: BMC Infect Dis. 2013 Nov 12;13:537. doi: 10.1186/1471-2334-13-537 (PMC3879221; doi:10.1186/1471-2334-13-537)
Supplement: Additional file 2 — The distribution of the number of control sequences clustering together with the patient’s own sequences in those patients whose sequences failed to form a monophyletic cluster. The number of clustering control sequences was either small (<20) or large (>50). We hypothesized that the former case is more likely to represent a transmission cluster, and used the criterion for the latter case in the identification of superinfection. Phylogenetic trees were constructed from sets of 150 sequences. [file 1471-2334-13-537-S2.pdf]

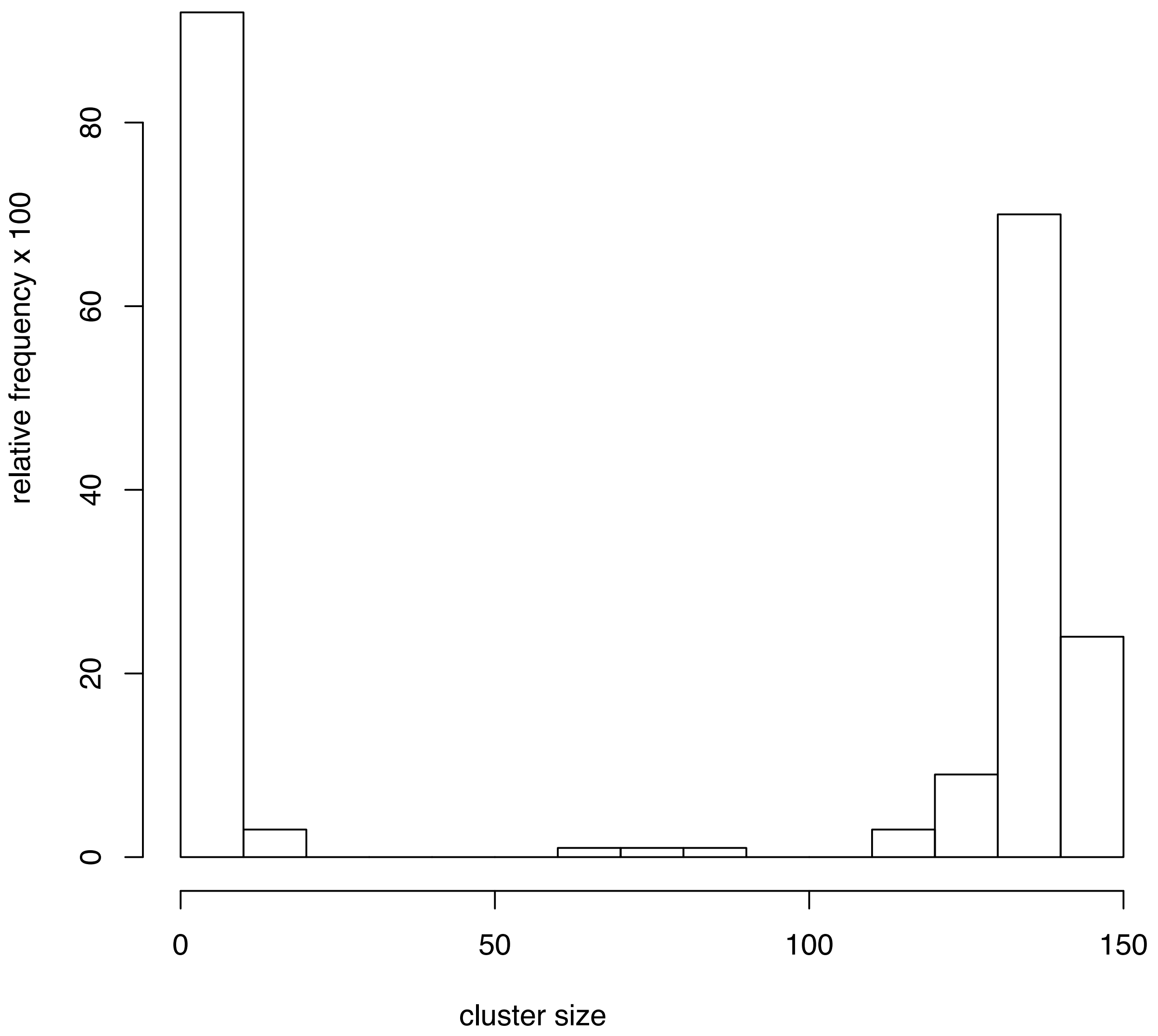

Distribution of the number of control sequences clustering
together with the patient's own sequences in those patients, whose sequences failed to form a monophyletic cluster.
