# Supplementary material for: Superinfection with drug-resistant HIV is rare and does not contribute substantially to therapy failure in a large European cohort
Source: BMC Infect Dis. 2013 Nov 12;13:537. doi: 10.1186/1471-2334-13-537 (PMC3879221; doi:10.1186/1471-2334-13-537)
Supplement: Additional file 3 — Table - The distribution of HIV sequences by subtype. Subtyping was carried out by the REGA subtyping tool [26,27]. [file 1471-2334-13-537-S3.pdf]

Distribution of HIV sequences by subtypes.

Subtyping was carried out by the REGA subtyping tool. In the cases where the REGA tool was not able to distinguish between a pure subtype and a subsubtype or circulating recombinant form, the latter is denoted in parentheses.

Subtype	#Sequences
HIV-1 Subtype B	11899
HIV-1 Subtype C	288
HIV-1 CRF 02_AG	263
HIV-1 Subtype A(A1)	235
HIV-1 Subtype F	161
HIV-1 Subtype G	92
HIV-1 Subtype A(01_AE)	85
HIV-1 CRF 06_CPX	26
HIV-1 Subtype G(02_AG)	18
HIV-1 Subtype D	16
HIV-1 Subtype J	12
HIV-1 Subtype A(A2)	11
HIV-1 CRF 12_BF	4
HIV-1 CRF 01_AE	5
HIV-1 CRF 11_CPX	4
HIV-1 CRF 13_CPX	2
HIV-1 CRF 10_CD	2
Undefined	693
