# Supplementary material for: Superinfection with drug-resistant HIV is rare and does not contribute substantially to therapy failure in a large European cohort
Source: BMC Infect Dis. 2013 Nov 12;13:537. doi: 10.1186/1471-2334-13-537 (PMC3879221; doi:10.1186/1471-2334-13-537)
Supplement: Additional file 4 — Additional Documentation - Supplementary text describing the verification of our bioinformatics pipeline and the approaches that did not succeed. [file 1471-2334-13-537-S4.pdf]

Additional Documentation

HIV superinfection is rare and does not contribute to drug resistance in the EUResist/Virolab cohort

1 Divergence based methods cannot reliably detect superinfection

The detection of superinfection involves an assessment whether the sequences obtained from a patient diverged from a last common ancestor within that patient, or several lineages were introduced by independent infection events. First we tested whether sequence divergence can be used to identify viral sequences of polyphyletic origin. In principle, a conservative divergence threshold could be estimated, based on the average within-host divergence between two time points: sequences more divergent than the threshold could be used

to identify superinfection. We performed a linear regression on pairwise phylogenetic distances of within-patient sequence pairs vs. the time between samples. While the regression confirmed a statistically significant increasing trend of sequence divergence over time, the large variation around the trend did not allow us to determine a clear threshold of within-patient sequence divergence over unit time (Supplementary Figure 1). The distributions of pairwise sequence distances calculated from interpatient and intrapatient sequence pairs also overlapped, and the distribution of intrapatient distances showed monotonous decrease, without a second minor peak that would be indicative of superinfections (Figure 3). It is therefore not possible to identify superinfection reliably based solely on sequence divergence. Moreover, the time of sampling is not a reliable indicator of the time span of evolution (number of generations) between two sequences, because virus strains archived in the latent reservoir can occasionally re-enter the replicating pool [Yin et al. 2012]. This issue will also prevent unambiguous determination of the order of entry of distinct strains in superinfected patients. Finally, the time scales of within-host and between-host evolution may overlap, i.e. divergent sequences sampled from one patient may have diverged within that patient (no superinfection), or may have diverged across a chain of transmissions. If a patient with long-time infection acquires a second virus, which had the same length of time for evolution and divergence since their most recent common ancestor, then we are unable to identify superinfection. Due to these potential problems we decided to evaluate superinfection based on clusters in a phylogenetic tree (as in [Lemey et al. 2005]) and not on divergence thresholds.

2 Preliminary analysis to the cluster based method

We started by building phylogenetic trees with RAxML due to its speed and ability to cope with huge number of sequences. Therefore as a first step we built RAxML trees from all 13816 sequences of 4425 patients. Because maximum likelihood reconstruction can be attracted to local optima and the large size of the tree decreases reliability, we built 100 replicates of random initialized RAxML trees. We analyzed each of the 100 trees to search for evidence of superinfection (as described above, and detailed in Methods) in each individual patient, and recorded, separately for each patient, the number of trees that indicated superinfection. The bimodal distribution of the number of trees that indicated superinfection in a given patient (Supplementary Figure 2.) was suggestive that the peak at 100 (including the cases that were identified as superinfected across all trees) may identify superinfections with high sensitivity. Using a conservative threshold, we selected for further analysis those patients (n=303) who were identified with putative superinfection in at least 50 replicate trees. As Bayesian inference is considered to be more thorough, but prohibitively slow for 4425 patients we analysed that 303 patients with MrBayes to gain further confidence in our results with RAxML. A separate phylogenetic tree was built for each of those 303 patients by the more exhaustive and much slower MrBayes software as follows. We selected a set of 150 control sequences for each patient from a massive local BLAST database of 70374 sequences such that each sequence of a patient was

matched and grouped with an equal number of similar sequences (see Methods and Figure 1.A). If the sequences of a patient include divergent lineages (from superinfection), this selection ensures that all lineages will be matched with control sequences of high similarity in the background set, while the tree size remains relatively small, which allows fast computation times.

Evaluating the trees with a threshold branch support of 0.95 confirmed 67 cases of putative superinfections, indicating that this subset was indeed enriched in such cases. To assess also the sensitivity of the quick maximum likelihood screening, we next analyzed a set of 100 randomly selected individuals who showed no evidence of superinfection in any of the RAxML trees. Performing the same analysis with MrBayes as described above, we found 3 individuals with evidence for superinfection in the Bayesian trees. This revealed that the screening procedure based on large maximum likelihood trees had insufficient sensitivity: a 3% false negative rate may have missed about 127 putative cases of superinfection in the 4121 patients who had not been subjected to detailed analysis in the preceding steps. We therefore decided to perform a detailed analysis of all patients separately.

In addition to the 67 patients who had evidence of superinfection based on the previous analysis, we selected another 103 patients randomly from the rest of the dataset, and used these 170 patients to calibrate the parameters of our final method as described in the main text. The outline of these steps are depicted on Additional File 5.

References

P. Lemey, S. Van Dooren, K. Van Laethem, Y. Schrooten, I. Derdelinckx, P. Goubau, F. Brun-Vézinet, D. Vaira, and A.-M. Vandamme. Molecular testing of multiple HIV-1 transmissions in a criminal case. *AIDS (London, England)*, 19(15):1649–58, Oct. 2005. ISSN 0269-9370. URL http://journals.lww.com/aidsonline/Abstract/2005/10140/Molecular_testing_of_multiple_HIV_1_transmissions.12.aspxhttp://www.ncbi.nlm.nih.gov/pubmed/16184035.

L. Yin, L. Liu, Y. Sun, W. Hou, A. C. Lowe, B. P. Gardner, M. Salemi, W. B. Williams, W. G. Farmerie, J. W. Sleasman, and M. M. Goodenow. High-resolution deep sequencing reveals biodiversity, population structure, and persistence of HIV-1 quasispecies within host ecosystems. *Retrovirology*, 9(1):108, Dec. 2012. ISSN 1742-4690. doi: 10.1186/1742-4690-9-108. URL http://www.ncbi.nlm.nih.gov/pubmed/23244298.

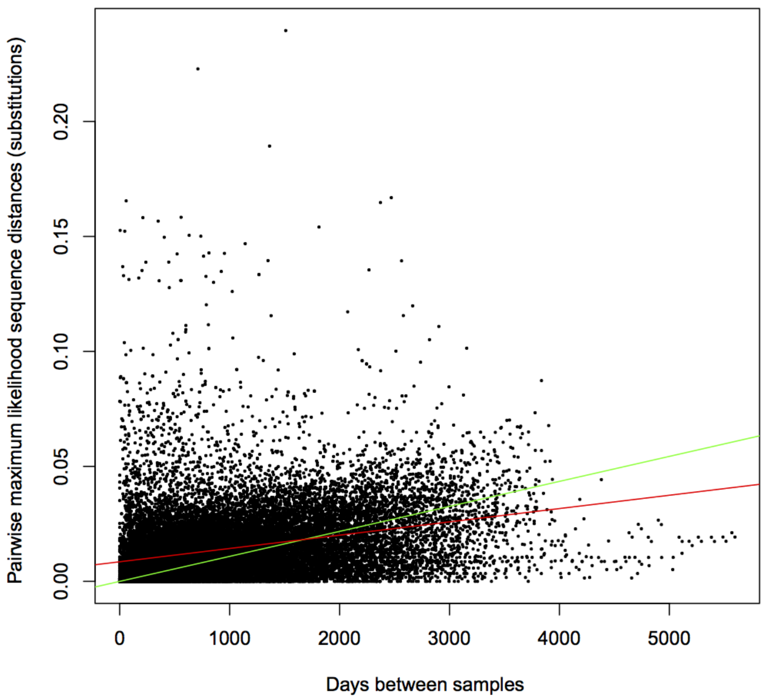

Figure 1: Supplementary Figure 1. - Pairwise distances of within-patient sequence pairs vs. time between samples. Pairwise sequence distances were calculated in a maximum likelihood framework under the GTR substitution model. The red line is the linear regression line not forced to pass through the origin ($p < 1 \times 10^{-10}$, slope $= 5.8 \times 10^{-6}$), while the green line is the linear regression line forced to pass through the origin ($p < 1 \times 10^{-10}$, slope$=1.09 \times 10^{-5}$). The large variation around the trend lines did not allow us to set a meaningful threshold on the rate of divergence to be used as a criterion for putative superinfection. Both regressions clearly overestimate divergence over the longest periods: these may represent slowly progressing or well-controlled infections with a slower pace of virus evolution.

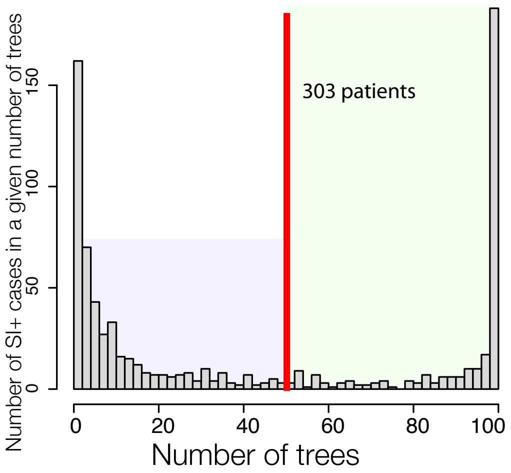

Figure 2: Supplementary Figure 2. - Preliminary analyses: results from 100 replicates of random initialized RAxML trees. As a first step we built 100 replicates of random initialized RAxML trees from all sequences of 4425 patients. The histogram shows the numbers of patients that were suspected for superinfection (SI+) based on the analysis of a given number of replicate trees. We selected those 303 patients that showed a putative superinfection in at least 50 trees for further analysis by MrBayes. Data from patients that were not suspected for superinfection in any trees are not shown.
