# Supplementary material for: Superinfection with drug-resistant HIV is rare and does not contribute substantially to therapy failure in a large European cohort
Source: BMC Infect Dis. 2013 Nov 12;13:537. doi: 10.1186/1471-2334-13-537 (PMC3879221; doi:10.1186/1471-2334-13-537)
Supplement: Additional file 5 — Figure - Chart of the whole phylogeny based analysis including the preliminary analyses. [file 1471-2334-13-537-S5.pdf]

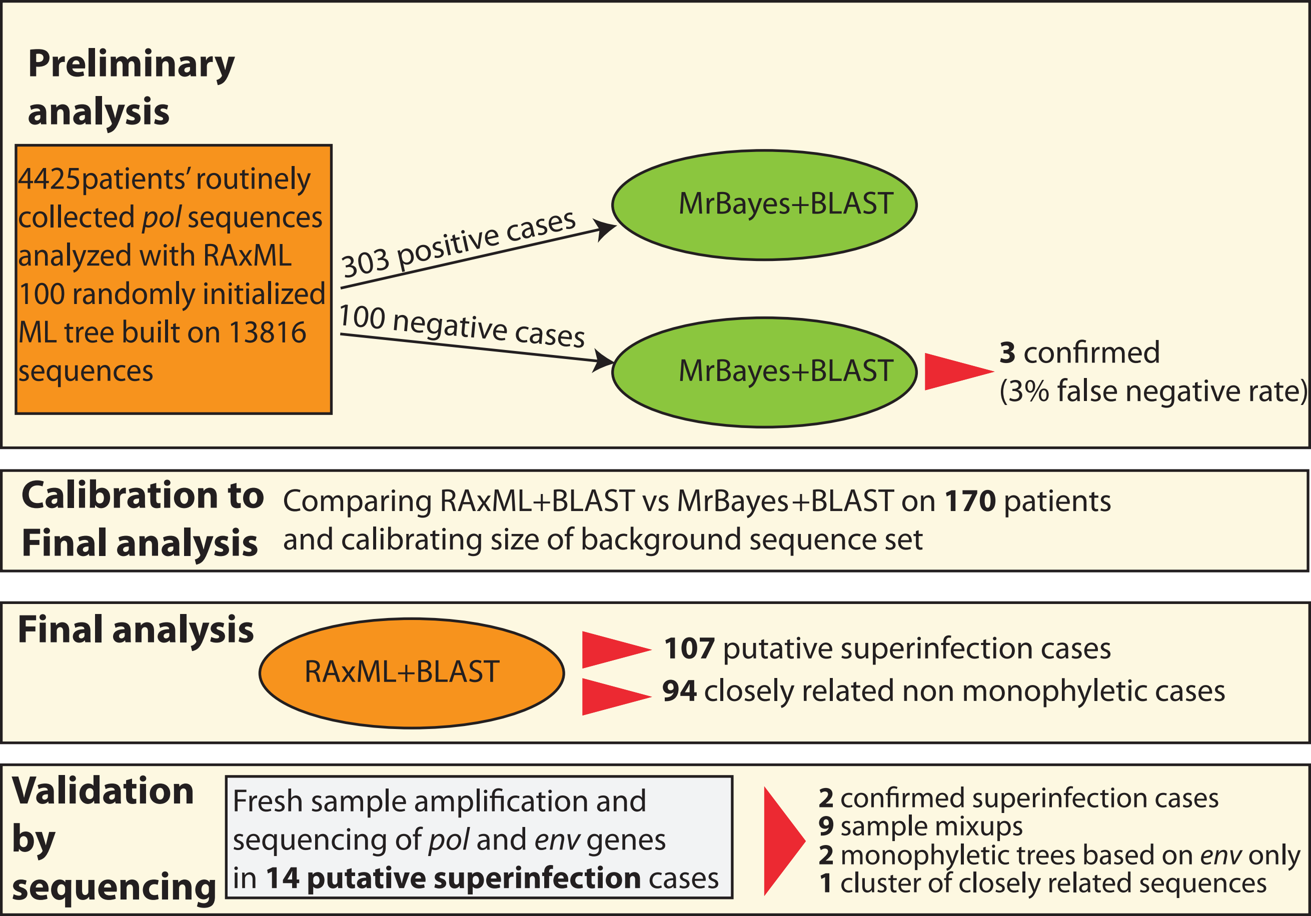
Preliminary analysis
4425patients' routinely collected *pol* sequences analyzed with RAxML 100 randomly initialized ML tree built on 13816 sequences
303 positive cases
MrBayes+BLAST
100 negative cases
MrBayes+BLAST
3 confirmed (3% false negative rate)
Calibration to Final analysis
Comparing RAxML+BLAST vs MrBayes+BLAST on **170** patients and calibrating size of background sequence set
Final analysis
RAxML+BLAST
107 putative superinfection cases
94 closely related non monophyletic cases
Validation by sequencing
Fresh sample amplification and sequencing of *pol* and *env* genes in **14 putative superinfection** cases
2 confirmed superinfection cases
9 sample mixups
2 monophyletic trees based on *env* only
1 cluster of closely related sequences
