# Supplementary figures and images for: Superinfection with drug-resistant HIV is rare and does not contribute substantially to therapy failure in a large European cohort
Source: BMC Infect Dis. 2013 Nov 12;13:537. doi: 10.1186/1471-2334-13-537 (PMC3879221; doi:10.1186/1471-2334-13-537)

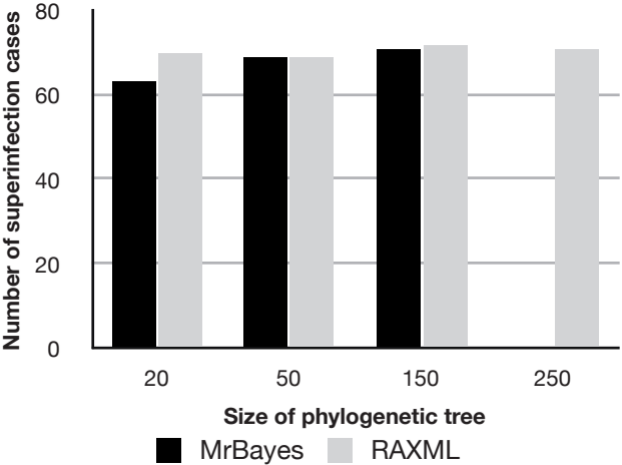

Supplement: Additional file 6 — The detection of superinfection is robust with respect to the number of control sequences in the trees. We performed both MrBayes and RAxML analyses with tree sizes of 20, 50, 150 and (only for RAxML) 250 sequences on a subset of the data (170 patients). Branches with a minimum support value of 60 or a minimum posterior probability of 0.95 were retained in the trees constructed with RAxML and MrBayes, respectively. The number of suspected patients was robust with respect to tree size. [file 1471-2334-13-537-S6.pdf]
